# Non-motor Symptoms in Patients with Parkinson’s Disease: A Cross-sectional Survey

**DOI:** 10.7759/cureus.3412

**Published:** 2018-10-04

**Authors:** Khushbakht Tanveer, Immad Attique, Waleed Sadiq, Arsalan Ahmad

**Affiliations:** 1 Neurology, Shifa International Hospital, Islamabad, PAK; 2 Internal Medicine, Shifa International Hospital, Islamabad, PAK

**Keywords:** parkinson disease, parkinsons, parkinsonism, parkinson\'s disease, non-motor symptoms frequency, early onset parkinson’s disease, frequency

## Abstract

Background

In multiple studies around the globe, non-motor symptoms (NMS) have been identified as a source of immense disability in patients with Parkinson’s disease (PD). However, there is a scarcity of data from Asia. This is the first study of the Pakistani population to assess the impact of NMS in PD on patients.

Objectives

To determine the frequency of NMS of PD in the Pakistani population and compare it with existing data.

Methods

In this cross-sectional survey, patient demographics were retrospectively collected from a tertiary care hospital neurology database. This study population comprised 97 patients at different stages of PD who presented to the neurology outpatient department. Disease severity was assessed using the Hoehn and Yahr scale. The NMS questionnaire was employed to identify the presence of NMS. Medical records were reviewed for demographic data and recent treatment history.

Results

The mean age was 67 years (76.3% of patients had adult onset PD and 23.7% had young onset PD). The NMS with the highest frequencies were nocturia (77.3%), urinary urgency (61.9%), constipation (59.8%), dementia (58.8%), insomnia (52.6%), and orthostatic hypotension (52.6%). The earliest manifestations of NMS were nocturia, forgetfulness, low mood, and orthostatic hypotension. Sleep abnormalities, falling episodes, and hallucinations are prevalent among patients with advanced disease.

Conclusion

There is a higher frequency of NMS present in the Pakistani population as compared to existing data in other populations.

## Introduction

Parkinson’s disease (PD) is a chronic and progressive neurodegenerative disorder. Insidious in onset, it is classically described as a triad of tremor, rigidity, and akinesia. According to recent data from the World Health Organization (WHO), the age-adjusted global incidence ranges from 9.7 to 13.8 per 100,000 population per year [1]. The lowest reported incidence was amongst Asians and African blacks whereas the highest was amongst whites. According to the Pakistan Parkinson’s society, an estimated 450,000 Pakistanis were afflicted with PD. This number was relatively low compared to values from India and China [2].

The years of healthy life lost because of disability per 100,000 people attributed to PD is reportedly 17.7 years [1]. This signifies PD is a major health issue given the disability it produces and the expense of rehabilitation and patient care. Recent advances in medical science have broadened our understanding of PD. PD encompasses several non-motor symptoms (NMS), further adding to patient disability. These NMS include neuropsychiatric manifestations, autonomic dysfunction, urinary complaints, sleep disorders, and sensory disturbances.

Shulman et al. reported neurologists failed to identify the presence of depression, anxiety, and fatigue during routine office visits of patients with PD more than half of the time and failed to recognize sleep disturbances in 40% of patients [3]. On the other hand, Gallagher et al. and Chaudhuri et al. found that patients frequently failed to link NMS with PD or were too embarrassed to discuss them [4-5]. The most frequently undeclared symptoms were delusions, daytime sleepiness, intense and vivid dreams, and dizziness [4-5]. Cheon et al. concluded that the average number of symptoms that patients knew to be associated with the disease were 5.2 ± 6.8, thus showing a lack of awareness regarding NMS amongst the patients [6]. Many of these potentially treatable symptoms were thus ignored, which emphasizes the need for physicians to take the initiative and actively screen for them.

Some recent studies discovered that a few NMS develop before the motor symptoms of PD manifest themselves. These NMS include olfaction, rapid eye movement (REM) sleep behavior disorder, fatigue, and depression [7]. These NMS could be used as preclinical markers of PD and lead to earlier diagnosis and treatment.

The objective of this study was to determine the frequency of the NMS of PD in the Pakistani population and compare it with existing data. This research was presented as a poster presentation in the EFNS-ENS Joint Congress of Neurology held in Istanbul, Turkey, from May 31, 2014, to June 3, 2014 (Paper Poster: K. Tanveer, I. Attique, W. Sadiq, F. Rao, A. Ahmed. Frequency Of Non-Motor Symptoms In Patients With Parkinson's Disease. Joint Congress of European Neurology; June 3, 2014).

## Materials and methods

Study approval, patient consent, and confidentiality

The study was approved by the institutional review board and ethics committee of Shifa International Hospital, Islamabad. All patients provided informed consent, and patient confidentiality was maintained by using patient registration numbers instead of patient names. This study was conducted in accordance with the Helsinki Declaration.

Inclusion criteria

This was an observational, cross-sectional study. All patients diagnosed with PD from 2010 to 2013 were accessed during follow-up visits to the neurology outpatient department of a tertiary care hospital in Pakistan. The patients completed the NMS questionnaire (NMS-Quest) during their visit. PD patients of all ages and in all stages of the disease were included. All patients were diagnosed by a specialist neurologist according to the UK Brain Bank Criteria for idiopathic PD [8]. For this study, the PD patient population was divided into two subgroups according to disease stage. One group comprised patients with a mild form of the disease (Hoehn and Yahr (H&Y) stages 1 and 2) and the other group were those with a severe form of the disease (H&Y stages 4 and 5).

Exclusion criteria

Patients with comorbidities that may introduce bias in the data were excluded. These comorbidities include thyroid disorder, men with benign prostatic hyperplasia, diagnosed psychiatric disorder, and heart disorder. Patients with type 1 diabetes and a glycosylated hemoglobin score >6 were excluded from the study as such patients may have symptoms of autonomic neuropathy that could introduce bias in the study. Patients diagnosed with dementia were excluded from the study due to the difficulty of these patients to correctly understand and answer the questionnaire. Patients with drug-induced Parkinsonism, vascular Parkinsonism, and atypical forms of Parkinsonism (such as progressive supranuclear palsy, multiple system atrophy, or corticobasal degeneration) according to accepted diagnostic criteria [9] were also excluded from the study.

Study tools

Disease severity was rated using the H&Y scale by a consultant neurologist. Patients were asked to complete the NMS-Quest [10], a self-completed patient questionnaire with 30 qualitative questions designed to identify the presence but not the severity of NMS. The NMS-Quest covers all important NMS of PD [11]. Patient files were used to obtain demographic data, treatment history, and comorbidities.

Statistics

The sample size was calculated using a sample size calculator for a proportion or descriptive study at www.openepi.com (OpenEpi Version 3) [12]. Population size was set as one million. Confidence limits were set at 5%. The anticipated percentage frequency was set as 97.3% [13]. The necessary sample size was calculated to be 70 to obtain a 99% confidence level.

Statistical analyses were performed using SPSS (SPSS Inc. Released 2007. SPSS for Windows, Version 16.0. Chicago, SPSS Inc.). Mean age and disease duration in each age group was calculated. To obtain prevalence for each NMS domain, the sum of the positive results was converted to a percentage based on the maximum number of responses in the domain.

## Results

In a sample of 97 PD patients, the mean age was 67 ± 10 and the mean disease duration was 7 ± 6 years. We noted that 62.9% (n = 61) of the patients were male and 37.1% (n = 36) were female. We found 23.7% (n = 23) of the patients were in H&Y stage 1, 25.8% (n = 25) were in H&Y stage 2, 26.8% (n = 26) were in H&Y stage 3, 15.5% (n = 15) were in H&Y stage 4, and 8.2% (n = 8) were in H&Y stage 5. NMS occurred in equal frequency among men and women. All patients were on at least one drug used for PD. According to our results, 4.1% (n = 4) of the patients were on one PD medication, 21.6% (n = 21) of patients were on two PD medications, 44.3% (n = 43) of the patients were on three PD medications, 26.8% (n = 26) of the patients were on four PD medications, and 3.1% (n = 3) of the patients were on five PD medications. We found 85.6% (n = 83) were taking levo-dopa, 71.1% (n = 69) were taking dopamine agonists, 42.3% (n = 41) were taking anti-cholinergics, 13.4% (n = 13) were taking catechol-O-methyltransferase inhibitors, 22.7% (n=22) were taking monoamine oxidase inhibitors, and 69.1% (n = 67) were taking amantadine.

**Figure 1:**
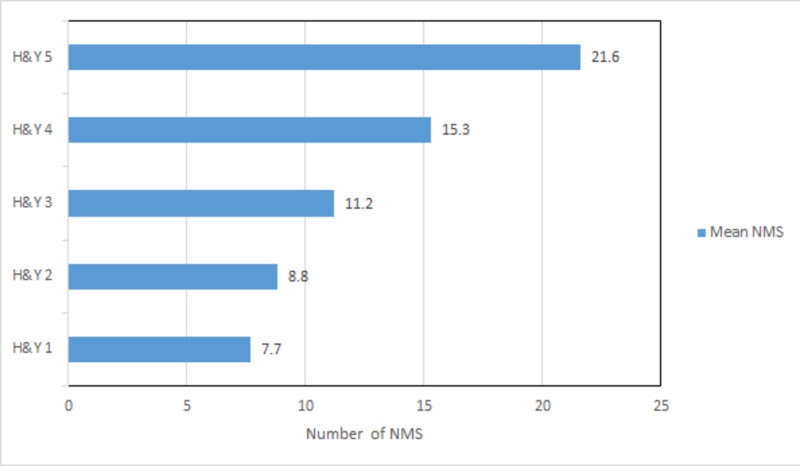
Average number of NMS present in different H&Y PD stages Abbreviations: NMS, non-motor symptoms; H&Y, Hoehn and Yahr; PD, Parkinson’s disease

Figure 1 shows the mean number of total NMS found in each disease stage. It demonstrates the average number of NMS increases with the disease severity; as the motor stage of the disease increases, the non-motor dysfunction also increases simultaneously. The mean total NMS for stages 1 and 5 were 7.7 and 21.6, respectively, showing an increase in non-motor dysfunction from stage 1 to 5.

Table 1 shows the frequency of each NMS found in the study population in descending order of frequency. The NMS with the highest frequency were nocturia (77.3%, n = 75), urinary urgency (61.9%, n = 60), constipation (59.8%, n = 58), dementia (58.8%, n = 57), insomnia (52.6%, n = 51), and orthostatic hypotension (52.6%, n = 51).

**Table 1:** Frequency of each non-motor symptom in decreasing order

Non-motor symptom	N	Percentage
Nocturia	75	77.3
Urinary urgency	60	61.9
Constipation	58	59.8
Reduced memory	57	58.8
Orthostatic hypotension	51	52.6
Insomnia	51	52.6
Low mood	50	51.5
Generalized body pains	46	47.4
Falling episodes	45	46.4
Loss of interest/apathy	41	42.3
Restless leg	41	42.3
Daytime somnolence	40	41.2
Anxiety	39	40.2
Weight loss	37	38.1
Excessive sweating	36	37.1
Sialorrhea	36	37.1
Dream reenactment	35	36.1
Impaired concentration	34	35.1
Vivid dream imagery	34	35.1
Leg swelling	31	32.0
Sensory or auditory hallucinations	29	29.9
Dysphagia	27	27.8
Tenesmus	25	25.8
Hyposmia	25	25.8
Bowel incontinence	23	23.7
Nausea/vomiting	23	23.7
Delusions	22	22.7
Double vision	13	13.4

To determine the symptoms of the disease appearing the earliest, we looked at the most frequently occurring symptoms in patients with a disease duration of less than one year (n = 10). We found that nocturia (nine patients), depression (seven patients), constipation (six patients), urgency (six patients), and orthostatic hypotension (five patients) were the earliest symptoms to appear in PD.

**Figure 2:**
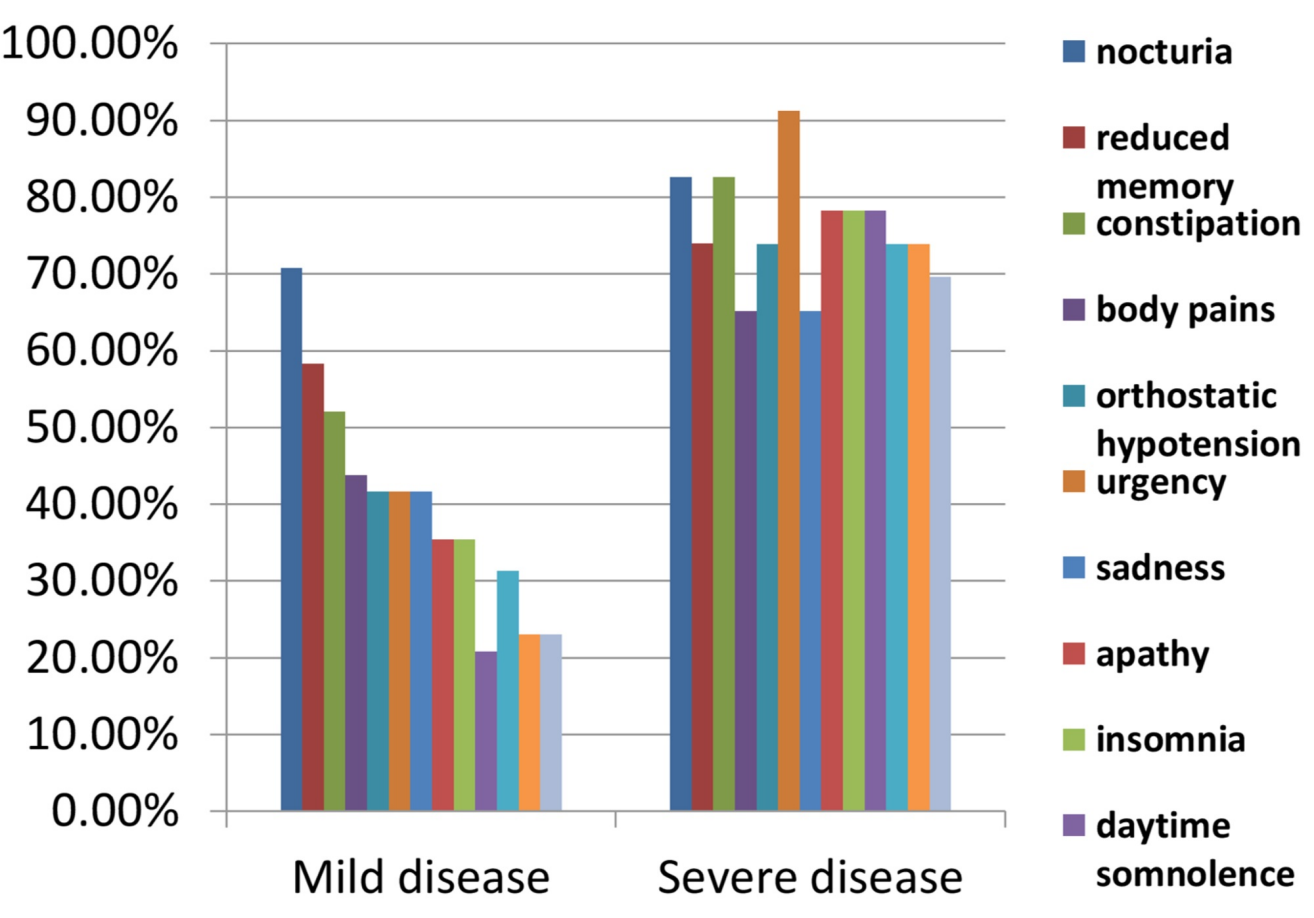
Comparison of frequency of commonest symptoms in mild and severe diseases

Figure 2 illustrates the most frequent symptoms found in the mild form (H&Y stages 1 and 2) of PD. It shows that autonomic dysfunction begins very early in PD, as illustrated by high percentages of urinary complaints, constipation, and orthostatic hypotension. The high frequency of memory dysfunction with the mild form of PD was an alarming feature of our population.

Figure 2 also illustrates that the symptoms common in the mild form of PD were also the most frequent features of severe forms of PD (H&Y stages 4 and 5). However, the domain of sleep disorders emerged as the most frequent feature of the severe form of PD if insomnia, vivid imagery, dream re-enactment, and daytime somnolence were collectively considered. Another unique feature of the severe form of PD was the high frequency of falling episodes. Our results showed that 73.3% of patients with falling episodes also had orthostatic hypotension.

## Discussion

To our knowledge, this is the first study on NMS prevalence in the Pakistani PD population. Our results are comparable to Li et al.who found a 100% prevalence rate of NMS in a cohort at a tertiary care hospital in China [14].

NMS-Quest demonstrated an average sensitivity of 63.4% and a specificity of 88.5% in comparison to the gold-standard evaluations during its validation [15]. However, the sensitivity showed a variable trend depending on the NMS under assessment. NMS-Quest is a simple tool that can be used in outpatient settings for the rapid screening of non-motor dysfunction in PD patients that would then require further evaluation of the symptoms identified using more sensitive methods. Most participants belonged to the elderly group and likely experienced a natural decline in sexual function. However, cultural barriers led to a marked reluctance by participants to respond to these questions, as had been faced by some previous studies on PD NMS [5]. Thus, we eliminated the category of sexual dysfunction from our data due to the poor response rate, reducing our data to responses to 28 out of the 30 NMS questions.

The most common NMS reported in our study were urinary urgency (77.3%), nocturia (61.9%), and constipation (59.8). These results were consistent with several international studies where nocturia, urinary urgency, constipation, and sadness were the most frequently reported NMS [16]. Reduced memory (58.8%), orthostatic hypotension (52.6%), insomnia (52.6%), and low mood (51.5%) were the other highly prevalent NMS reported in our study, mirroring the results found elsewhere [4]. We observed a much higher frequency of these symptoms in our population compared to other studies, consistent with the results of a similar study done by Cosentino et al. [17]. Due to a lack of regional data, we need further data to determine if Asians are more prone to non-motor dysfunction. Another possible reason for this discrepancy was the lack of awareness amongst physicians about NMS, so they are left untreated. With advancing H&Y stage, the total number of NMS increases, as was seen by Cosentino et al. [17].

In general, the prevalence rate of urinary dysfunction amongst PD patients ranges from 37% to 70% [18]. Winge et al. demonstrated using single-photon emission computed tomography (CT) imaging that the severity of urinary dysfunction correlated with the relative degeneration of the dopamine-dependent caudate nucleus [19]. Lewy bodies in the autonomic nervous system of patients with advanced PD can account for urinary sphincter dysfunction and rare cases of hyporeflexia of the detrusor muscle [18]. In our study, 77.3% of patients complained of nocturia while 61.9% of the patients had urinary urgency. Considering the difference in average disease duration, our results closely match Martinez-Martin et al. who found that 59% of their patients had some sort of urinary complaint [16]. According to our data, 59.8% of the patients had constipation; these results closely resembled those of Azmin et al. who reported gastrointestinal complaints in 61.9% of their study population [13]. Azmin et al. also concluded that there is a higher tendency in the Asian PD population for constipation with poverty and diet as the most likely reasons [13].

Orthostatic hypotension was found in 52.6% of our patient population. Damage to the postganglionic sympathetic efferents, reduced sympathetic noradrenergic innervations of the left ventricular myocardium, and arterial baroreflex failure account for orthostatic hypotension in PD [20]. One study recommended screening for orthostatic hypotension in all PD patients in different positions to ensure early detection, as it can be easily treated by drug therapy [21].

We observed a marked increase in falling episodes in the severe form of PD. As orthostatic hypotension worsens, it leads to significantly higher frequencies of falls; 73.3% of patients with falling episodes also had orthostatic hypotension. Since these patients were in stages 4 and 5 of PD, they experienced balancing problems, freezing gait, daytime sleepiness, and reduced cognition. These combined to cause an increased frequency of falls [22]. Thus, fall precautions should be considered in patients with severe forms of PD.

Our results show that memory reduction begins with the mild form of the disease with 58.3% frequency at stages 1 and 2 and increasing in frequency to 73.9% in later stages. Zarei et al. described cortical thickness, cortical folding, and gray matter volume representing neurodegenerative changes in PD [23]. The progression of disease was related to the ascending spread of α-synuclein deposition (Lewy bodies and neurites) from the lower brainstem nuclei to cortical areas, leading to cognitive dysfunction and memory loss, following the stages proposed by Braak et al. [24].

Limitations

Our results may be biased as it is a questionnaire-based study. The validation study of the questionnaire indicated that for some manifestations such as somnolence, olfactory loss, and apathy, the sensitivity of the questionnaire is suboptimal [13]. Additionally, the study sample was small.

## Conclusions

Every patient in our study had at least one NMS present. There was a higher frequency of NMS present in our population as compared with existing data; this suggests South Asians are more prone to non-motor dysfunction in PD. There is a need to conduct more studies in the South Asian population on a larger scale that would look at the frequency of NMS in PD.
